# Accelerated Immunodeficiency by Anti-CCR5 Treatment in HIV Infection

**DOI:** 10.1371/journal.pcbi.1000467

**Published:** 2009-08-14

**Authors:** Ariel D. Weinberger, Alan S. Perelson, Ruy M. Ribeiro, Leor S. Weinberger

**Affiliations:** 1 Biophysics Graduate Group, University of California, Berkeley, California, United States of America; 2 Theoretical Biology and Biophysics, Los Alamos National Laboratory, Los Alamos, New Mexico, United States of America; 3 Department of Chemistry and Biochemistry, University of California, La Jolla, California, United States of America; Utrecht University, Netherlands

## Abstract

In 50% of progressing HIV-1 patients, CXCR4-tropic (X4) virus emerges late in infection, often overtaking CCR5-tropic (R5) virus as the dominant viral strain. This “phenotypic switch” is strongly associated with rapidly declining CD4^+^ T cell counts and AIDS onset, yet its causes remain unknown. Here, we analyze a mathematical model for the mechanism of X4 emergence in late-stage HIV infection and use this analysis to evaluate the utility of a promising new class of antiretroviral drugs—CCR5 inhibitors—in dual R5, X4 infection. The model shows that the R5-to-X4 switch occurs as CD4^+^ T cell activation levels increase above a threshold and as CD4^+^ T cell counts decrease below a threshold during late-stage HIV infection. Importantly, the model also shows that highly active antiretroviral therapy (HAART) can inhibit X4 emergence but that monotherapy with CCR5 blockers can accelerate X4 onset and immunodeficiency if X4 infection of memory CD4^+^ T cells occurs at a high rate. Fortunately, when CXCR4 blockers or HAART are used in conjunction with CCR5 blockers, this risk of accelerated immunodeficiency is eliminated. The results suggest that CCR5 blockers will be more effective when used in combination with CXCR4 blockers and caution against CCR5 blockers in the absence of an effective HAART regimen or during HAART failure.

## Author Summary

HIV has caused over 30 million deaths. The virus is so fatal because it infects and depletes CD4^+^ T cells, “helper” immune cells critical for orchestrating and stimulating the overall immune response. No one understands why, in about 50% of HIV infections, a more deadly strain emerges late in infection. The new HIV strain, known as X4, differs from its predecessor, known as R5, because X4 only infects CD4^+^ T cells displaying the receptor CXCR4, while R5 only infects CD4^+^ T cells displaying the receptor CCR5. Because CXCR4 and CCR5 are found on different CD4^+^ T cells, X4 depletes a second set of critical immune cells, accelerating immunodeficiency and death. Recently, the FDA began approving drugs that selectively block R5, and some researchers have touted anti-R5 therapy alone as a potentially safer alternative to current anti-HIV drugs. But an open question is whether anti-R5 treatments push HIV toward the more deadly X4 variant earlier. To understand how X4 emerges and how anti-R5 treatments affect X4, we apply a combination of mathematical analysis and simulation. An important medical result of our work is that anti-R5 treatment alone can accelerate X4 emergence and immunodeficiency. Our results suggest that anti-R5 treatment only be used with anti-X4 treatment or anti-HIV drug “cocktails,” which combat R5 and X4 equally.

## Introduction 

Left untreated, human immunodeficiency virus type-1 (HIV) generally targets and severely depletes a patient's CD4^+^ T cells over a period of up to 15 years, with a median AIDS onset time of 9.8 years [1]–[4]. HIV's infection of a CD4^+^ T cell begins when HIV's outer envelope protein gp120 binds to a CD4 receptor and subsequently binds to one of two chemokine coreceptors, CCR5 or CXCR4 [5],[6]. Viral-coreceptor binding exposes a second viral envelope protein, gp41, which mediates fusion of the viral and target-cell membranes, allowing HIV to inject its retroviral material into the cell. HIV strains that use CCR5 as a coreceptor are termed R5 viruses, while those that bind CXCR4 are called X4 viruses.

R5 virus is predominant during early infection where X4 virus has rarely been observed, independent of the route of viral transmission [5], [7]–[9]. Importantly, X4 *alone* is generally unable to infect humans: individuals homozygous for a 32 base-pair deletion in CCR5, CCR5Δ32, are almost entirely immune to HIV [5]. However, in approximately 50% of progressing HIV patients, a ‘phenotypic switch’ occurs wherein X4 virus emerges late in infection, overtaking R5 virus as the dominant viral strain. The R5-to-X4 switch is strongly associated with a poor clinical prognosis for the patient: it occurs with a steep loss in CD4^+^ T cell counts and accelerated AIDS onset.

The mechanisms causing R5's early dominance and the subsequent R5-to-X4 switch are poorly understood, however multiple lines of evidence suggest that CCR5's higher cell-surface density on activated and recently activated memory CD4^+^ T cells enable R5 to infect more of this crucial cellular population than X4. CCR5's cell-surface density has been shown to determine the efficiency of R5 infection [10], possibly because multiple CCR5 receptors act in a cooperative, concentration-dependent manner to facilitate infection [11]. R5 virus' level of infection is thus highest among CD62L^−^ effector memory CD4^+^ T cells [12], where CCR5's cell surface density is highest. CXCR4's cell-surface density is similarly positively correlated with X4's emergence [13], but CXCR4's per-cell density on memory CD4^+^ T cells is lower than that of CCR5 [14], giving R5 an advantage on these cells. On dually-positive CCR5^+^, CXCR4^+^ CD4^+^ T cells, the coreceptors compete for association with CD4 [15], which should lend R5 an advantage given CCR5's higher per-cell surface density on dually-positive cells [14].

Thus, R5 virus' early advantage may stem from CCR5's greater *per-cell* surface density on activated and recently activated ‘effector’ memory CD4^+^ T cells [14],[15]. These ‘effector’ memory CD4^+^ T cells are the crucial virion-producing populations as evidenced by snapshots taken during SIV infection, which show approximately five times as many virions surrounding infected, activated effector memory CD4^+^ T cells as around infected, quiescent CD4^+^ T cells [16]. Moreover, Li et al. show that CD4^+^ T cells positive for Ki67 (a marker that is displayed after late G1 cell-cycle progression and indicates T cell ‘activation’) produce over 90% of the virions during the chronic phase of SIV infection [17]. This may also explain why X4 has trouble initiating infection when R5 virus is absent: CXCR4's per-cell density on the most crucial memory CD4^+^ T cell population is simply too low [14].

*The perplexing question underlying the R5-to-X4 phenotypic switch is therefore: how does a switch to X4 occur if R5 virus is simply better at infecting memory CD4^+^ T cells?*

Since the R5-to-X4 switch only occurs during late infection, it is reasonable that there exists an early selection pressure in favor of R5 virus, which is mitigated over the course of infection. In support of this hypothesis, Ribeiro and colleagues recently proposed the idea that increasing target-cell activation over the course of dual infection causes X4 to eventually outcompete R5 [18].

A critical prediction of the Ribeiro model is that CCR5 blockers (small-molecule pharmaceuticals that bind CCR5 and thereby obstruct R5 virus' ability to infect a CD4^+^ T cell) successfully reduce overall viral loads, decrease cellular activation levels, and inhibit X4 emergence. This prediction is critical since a central question is whether CCR5 blockers lend X4 virus an advantage and promote clinically deleterious switches to X4 during dual R5 and X4 infection. However, *in vivo* trials of the CCR5 inhibitors *CMPD 167* and *maraviroc* showed CCR5 blockers actually increasing X4 viral loads and decreasing R5 viral loads (approximately reciprocally) in dually-infected patients [19],[20].

Given the recent CCR5 clinical trial data, we analytically probed how changing target cell activation levels could produce a switch and whether such models could account for documented increases in X4 viral load after anti-CCR5 treatment. Our model builds upon [18], but in our generalized setup the R5-to-X4 switch can occur even if the fraction of activated naïve CD4^+^ T cells increases at a slower rate than the fraction of activated memory CD4^+^ T cells. In this more general setting, we rigorously show how the R5 to X4 switch occurs and find that CCR5 blockers often do accelerate X4's emergence and attendant immunodeficiency. Fortunately, the results also show that when CXCR4 inhibitors or HAART are given along with CCR5 inhibitors, X4 emergence is unlikely to be accelerated and is instead often delayed.

## Models

In the following three models, all variables are capitalized and represent concentrations per microliter (1/µl). Specifically, in Model 1, *T* represents the concentration of uninfected CD4^+^ T cells, and (without loss of generality) is given an initial value of 1000 CD4^+^ T cells/µl. In Models 2 and 3, *T* is split into uninfected naïve (*N*) and memory (*M*) subpopulations, each with an initial value of 500 CD4^+^ T cells/µl. I_4_ and I_5_ reflect the concentrations of *abortively, latently, and productively* infected CD4^+^ T cells by X4 and R5, respectively, and in Model 3, we analogously define *N_4_, M_4_, M_5_* (see below). *V_4_* and *V_5_*, each given initial values of 1000 virions/ml, represent X4 and R5 viral load concentrations.

Defining the parameters, *λ* is the rate of thymus production of CD4^+^ T cells and has units cells/(µl•day), *k_4_* and *k_5_* are the respective infection rate coefficients for X4 and R5 infection of CD4^+^ T cells and have the units µl/(virions•day). All remaining parameters have units 1/day. These include: *d_T_*, the death rate of uninfected CD4^+^ T cells in Model 1, set to *λ/T_0_* to allow for steady-state pre-infection, and *d_n_* and *d_m_* the analogous death rates of uninfected CD4^+^ T cells in Models 2 and 3, also set so that equilibrium exists pre-infection. Additionally, *δ* is the death rate of infected CD4^+^ T cells, *p* is the rate of viral production by *activated* infected cells, and *c* is the viral clearance rate. *a_n_* and *a_m_* are required to satisfy Equation 2 (below) and represent the fractions of *activated* naïve and memory CD4^+^ T cells as a function of CD4, the total number of uninfected and infected CD4^+^ T cells per microliter. Thus, in Models 1 and 2, CD4 = *T+I_4_+I_5_*, and, analogously, in Model 3 CD4 = *N+M+N_4_+M_4_+M_5_*. Since the total concentration of CD4^+^ T cells changes over time, *a_n_* and *a_m_* vary over the course of infection.

Because over 99% of infected cells are defectively infected [21] and because such non-productively infected cells are indistinguishable from uninfected cells, we make the simplifying assumption that *a_n_* and *a_m_* also approximate the fractions of *infected* naïve and memory CD4^+^ T cells that are activated. Thus, in Models 1 and 2, *a_n_I_4_* and *a_m_I_5_* represent the concentrations of *activated* X4 and R5 infected cells, respectively. Analogously, in Model 3, *a_n_N_4_, a_m_M_4_*, and *a_m_*M_5_ represent the concentrations of *activated* X4-infected naïve, X4-infected memory, and R5-infected memory CD4^+^ T cells, respectively. In our models, it is only these activated subpopulations of infected cells that produce virions. We thus multiply the concentration of activated infected cells (e.g., *a_m_*M_5_) by *p*, the rate of viral production (per-day) from a *productively*-infected (i.e., activated and infected) cell, yielding the respective total concentration of virions produced each day by a given infected cell type.

### Model 1: Single Target Cell Compartment

We first extended the basic model of viral dynamics [1],[2] to two viral strains, to test whether this simplified, one-compartment model can generate a representative R5-to-X4 switch.
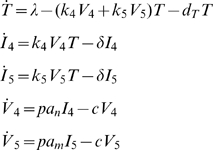
(Model 1)Here we make X4's viral production dependent on the fraction of activated naïve CD4^+^ T cells *a_n_*, but not on *a_m_*. One reason for this simplification is that R5 out-competes X4 for dually-positive memory CD4^+^ T cells [22]. Furthermore, the vast majority of CXCR4-positive T cells are in the naïve subset, where CXCR4's cell surface density is also highest [14]. Since Model 1 lumps all CD4^+^ T cells into a single target-cell compartment, and because across all lymphocytes CXCR4's median per-cell surface density is almost three times as high as that of CCR5 [14], we also assume *k_4_*>*k_5_*. As above, this does not imply that X4 *productively* infects more target cells than R5 at the beginning of infection, since very few naïve cells are activated early in infection [23]. Importantly, given the simplifications employed, the purpose of Model 1 is not to represent the actual dynamics of coreceptor tropism in HIV infection, but to rigorously explore an activation-based R5 to X4 switch in the simplest setting.

### Model 2: Two Target Cell Compartments

To account for the fact that in reality naïve and memory CD4^+^ T cells are disjoint target cell compartments, we subsequently build upon Model I and divide T into N and M.
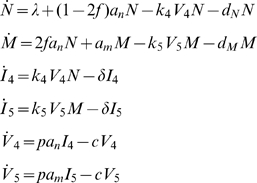
(Model 2)The equations in this system are analogous to those in Model 1 but the uninfected CD4^+^ T cell population is now split into uninfected naïve (N) and memory (M) subpopulations. Additionally, *f* is defined to be the fraction of naïve cells activated via the conventional Ag-TCR interaction, which divide and differentiate into CD45RO^+^ memory cells. The rest of the activated cells are assumed to have been upregulated via cytokines or other Ag-TCR independent processes and thus remain phenotypically naïve (CD45RA^+^) [24]–[26]. We note that non-Ag mediated activation of naïve CD4^+^ T cells is not absolutely necessary for our models' primary conclusions of strain coexistence and phenotypic switching at clinically-representative time-points (i.e., 3–6 years post-infection); we include this activation term for the added realism it brings to the model.

In Model 2, X4 is only able to infect naïve CD4^+^ T cells, a simplification we employ because of the data in [14] showing that the per-cell density of CCR5 is significantly higher than that of CXCR4 on memory CD4^+^ T cells. Moreover, a recent paper finds that on dually-positive CXCR4^+^, CCR5^+^ CD4^+^ T cells, R5 generally outcompetes X4 [22], arguably because of CCR5's higher surface density [15]. Finally, naïve CD4^+^ T cells have been found to be preferentially depleted during X4 infection [27].

### Model 3: Two Target Cell Compartments with Viral Competition

Because in practice X4 actually infects both naïve and memory CD4^+^ T cells, in our final model, Model 3, we extend the two-compartment setup of Model 2 to allow X4's infection of memory CD4^+^ T cells:
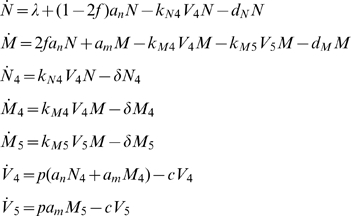
(Model 3)In this model, k_N4_ and k_M4_ are the infection rate coefficients of X4 on naïve (N) and memory (M) CD4^+^ T cells, respectively, and k_M5_ is the infection rate coefficient of R5 on memory CD4^+^ T cells. k_N4_, k_M4_, and k_M5_ all have units µl/(virions•day). *N_4_* and *M_4_* are the concentrations of *abortively, latently, and productively* infected naïve and memory CD4^+^ T cells, respectively, by X4 virus, and M_5_ is the concentration of *abortively, latently, and productively* infected memory CD4^+^ T cells by R5 virus. All other parameters, variables, and initial conditions have been defined above. Because CCR5 is far more strongly expressed on memory CD4^+^ T cells than is CXCR4 [14], we set k_M5_≫k_M4_. Conversely, CXCR4 is more highly expressed on naïve CD4^+^ T cells than it is on memory CD4^+^ T cells [14], making k_N4_≫k_M4_.

#### Generalized conditions for a_n_ and a_m_

HIV is associated with increasing levels of CD4^+^ T cell activation [23],[28],[29],[30]. Curve fitting *in vivo* data from [23], Ribeiro et al. [18] found that the fractions of phenotypically-activated (Ki67^+^) naïve (*a_n_*) and memory CD4^+^ T cells (*a_m_*) have the following inverse relationships to the total CD4^+^ T cell count per microliter (denoted CD4 in the equations below):
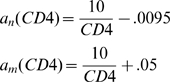
(1)


Rather than restrict ourselves to an analysis based on Eq. (1), we only assume that *a_n_* and *a_m_* obey three general conditions for all CD4^+^ T cell counts:
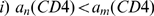


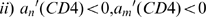
(2)

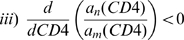
In other words, i) the fraction of activated cells is assumed to always be higher among memory CD4^+^ T cells than among naïve CD4^+^ T cells, ii) both fractions are assumed to be increasing as CD4^+^ T cell counts decline, and iii) as CD4^+^ T cells are depleted, the fraction of activated naïve CD4^+^ T cells increases *relative* to the fraction of activated memory CD4^+^ T cells. Importantly, the relative fraction *a_n_/a_m_* can increase even when *a_n_* increases at a slower rate than *a_m_* in response to CD4^+^ T cell decline.

## Results

### A Single Compartment Model Generates an R5 to X4 Switch without Coexistence

**Figure 1:**
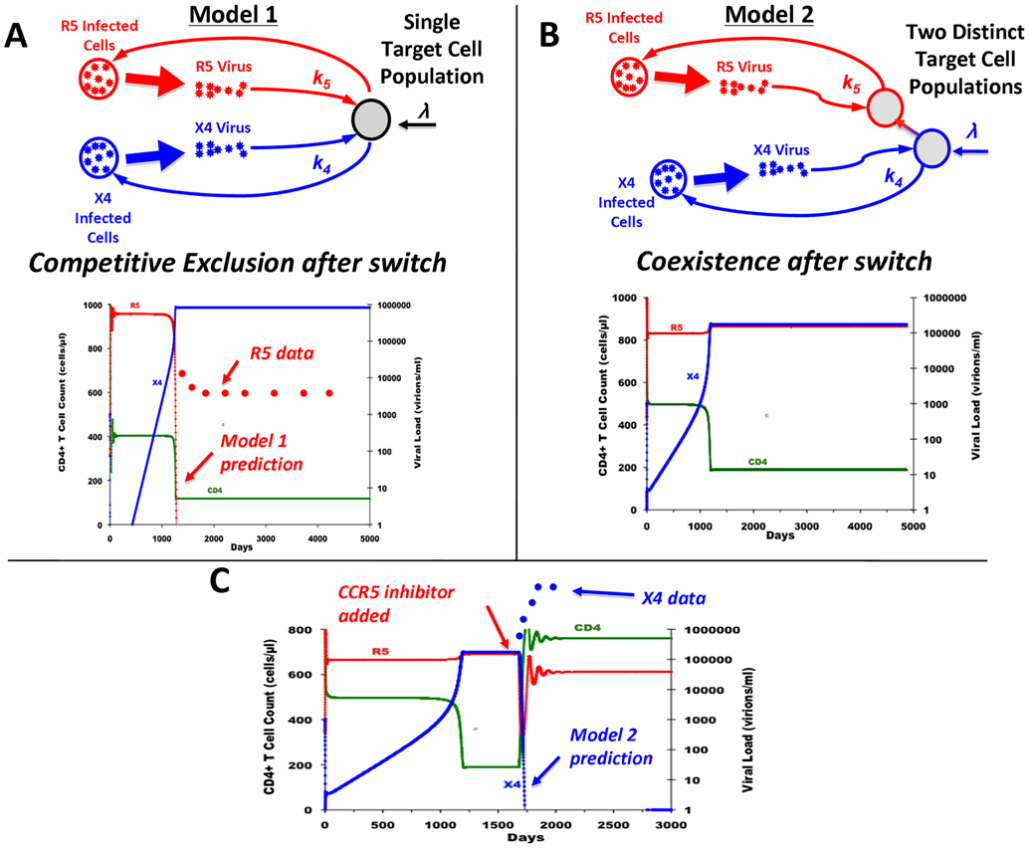
Models assuming a single target-cell population for X4 and R5, and models where X4 and R5 infect distinct populations cannot account for X4 and R5 data. (A) A pedagogical model (schematic and numerical simulations) that is oversimplified to account for only a single target-cell population generates a competitively exclusive R5-to-X4 switch where R5 virus is cleared (i.e. goes extinct) following the switch, contrary to *in vivo* data [32]. The model simulates a “phenotypic” switch occurring at a clinically representative time of 3–4 years post HIV-1 infection, and, notably, yields a concomitant decline in CD4^+^ T cell counts. The parameters used are *λ* = 33 cells/(µl•day), *c* = 23/day, *p* = 5750/day, *δ* = 0.7/day, *k_4_* = 5•10^−4^ µl/(virions•day), and *k_5_* = 10^−4^ µl/(virions•day). (B) A model restricting R5 and X4 to disparate target cell compartments can generate a clinically representative R5 to X4 switch over a large parameter regime and also exhibit coexistence of R5 and X4 post-switch. However, as shown in (C), such models cannot account for *in vivo* data showing that R5 inhibitors increase X4 levels [19]. In (C), we apply a CCR5 blocker with a drug efficacy of 0.9, starting at t = 180 days. The model restricts R5 and X4 to independent target cell compartments, and, given the absence of viral competition, always generates strong suppression of X4 in response to CCR5 inhibition. Simulations in (B) and (C) are shown for a representative parameter regime: *λ* = 33 cells/(µl•day), *c* = 23/day, *p* = 2000/day, *f* = 0.8, *δ* = 0.5/day, *k_4_* = 0.0012 µl/(virions•day), and *k_5_* = 0.0034 µl/(virions•day).

In single target-cell compartment susceptible-infectious (SI) models such as Model 1, the ecological principle of *competitive exclusion* generally applies [31]. Thus, while Model 1 can produce an R5 to X4 switch in a clinically representative timeframe, it necessarily manifests competitive exclusion (Fig 1a). The lack of steady-state coexistence in Model 1 is significantly different from data, which show long-term coexistence of R5 and X4 variants in post-switch individuals [32]. Moreover, X4's emergence late in infection—well into quasi-steady state—is very difficult to achieve in this single compartment framework, because X4 could have been rendered extinct via *competitive exclusion* prior to the late-stage switch (Weinberger and Perelson, *manuscript in preparation*).

### Two Target Cell Populations Can Produce R5 and X4 Coexistence

In order to prevent the species with the higher effective reproductive ratio from dominating *exclusively*, which contradicts observed results [32], Model 2 splits the target cell population into naïve and memory CD4^+^ T cells, and, for simplicity, assumes that X4 solely infects naive cells and that R5 only infects memory cells. The dual-target cell compartment nature of Model 2 makes coexistence possible (Weinberger and Perelson, 2009, *manuscript in preparation*). Thus, while Model 2 can also produce an R5-to-X4 switch at a clinically representative time, it is able to maintain R5 and X4 coexistence post-switch (Figure 1B).

However, CCR5 inhibition cannot produce a transient increase in X4 (Figure 1C). This is because in models that restrict X4 and R5 to infecting distinct target cell populations (e.g., Model 2), X4 does not infect any of the (memory) CCR5^+^ T cells that are made refractory to R5 infection by CCR5 inhibition. Quantitatively, Eq. (2) stipulates a_n_′(CD4)<0, so when CCR5 is inhibited and memory CD4^+^ T cell counts rise, *a_n_* decreases and the rate of viral production from an X4-infected cell (p*a_n_) is lowered. Due to the lack of competition, the number of X4-infected cells does not increase to compensate for the decreased per-cell virion production rate, so X4 viral loads decrease (Figure S1). This result is in contrast to recent studies on dually-infected *rhesus macaques* and humans, which demonstrate clear increases in X4 virus after R5 virus is selectively suppressed through the use of a CCR5 inhibitor [19],[20]. To produce a temporal X4 increase upon R5 inhibition and to maintain coexistence in contradistinction to Model 1, we need a multi-compartment model where X4 infects both naïve and memory CD4+ T cells.

### Two Target Cell Compartments with Viral Competition Allow Coexistence and Match Existing Data

**Figure 2:**
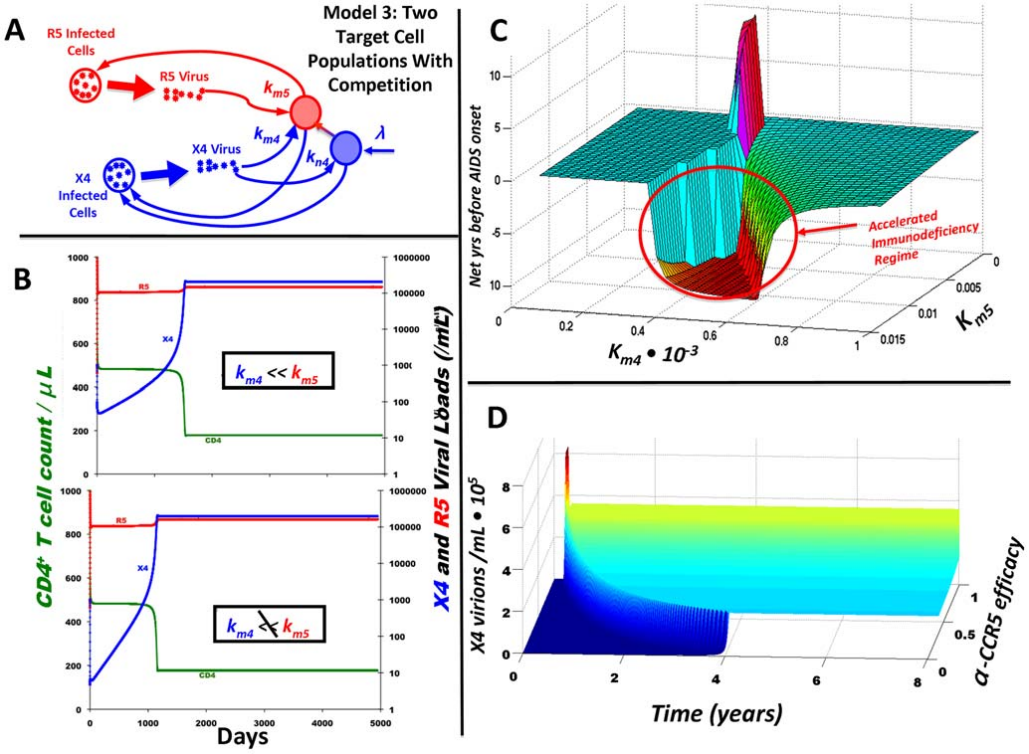
A model with two target-cell populations and viral competition in one of these populations matches *in vivo* data and predicts that R5 inhibitors accelerate AIDS onset. (A) A schematic of Model 3, a competitive model with two target-cell populations. (B) The model exhibits a coreceptor switch at approximately 1000–1400 days post-infection in two types of parameter regimes: the “non-competitive regime” in (B, upper panel) and the “competitive regime” (B, lower panel). For the “non-competitive regime,” parameter values are: *λ* = 33 cells/(µl•day), *c* = 23/day, *p* = 2100/day, *f* = 0.8, *δ* = 0.5/day, *k_N4_* = 0.00108 µl/(virions•day), *k_M4_* = 4•10^−5^ µl/(virions•day), and *k_M5_* = 0.0068 µl/(virions•day), while in the “competitive regime” we change *k_M4_* to 5•10^−4^ µl/(virions•day) and *k_N4_* to 0.001 µl/(virions•day) (decreased in order to keep X4 in check). (C) The net effect of a CCR5 inhibitor with 80% efficacy on the time to AIDS across different *k_M4_* and *k_M5_* levels (i.e. different competitive and non-competitive regimes). AIDS onset is defined as the time at which CD4^+^ T cell counts fall below 200 cells/µl: negative values represent accelerated times to AIDS-onset relative to no treatment. R5 inhibitors clearly accelerate AIDS-onset for a large fraction of parameter space. (D) Time-dependency of X4 emergence as a function of CCR5 inhibitor efficacy (α-CCR5 efficacy) in the “competitive regime.” Increased CCR5 inhibitor efficacy accelerates X4 emergence and increases X4's viral set point.

In Model 3, our final and most biologically detailed model, we include naïve and memory CD4^+^ T cell compartments. Since CXCR4 is found on a large number of memory CD4^+^ T cells, we allow for X4's infection of memory as well as naïve CD4^+^ T cells (Figure 2A). Thus, Model 3 serves as a union of the two previous models: it includes the X4 and R5 strain competition found in Model 1 and it also includes the separate target cell compartments of Model 2, which allowed for X4's persistence prior to a switch and the coexistence of strains afterward. Model 3 produces X4-to-R5 switches at clinically representative times of 1000–2000 days and also maintains coexistence post-switch in two types of parameter regimes, the “non-competitive” and “competitive” regimes, whose distinctions are elaborated upon below (Figure 2B).

Given that X4 and R5 viruses can coexist in the disjoint two-compartment model, it is reasonable to conjecture that coexistence is a feature of this extended model as well. To show this formally, we define *R_eff4_* and *R_eff5_* to be the *effective* reproductive ratios of X4 and R5 virus, respectively, which are given by:
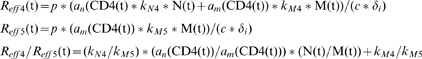
(3)The *effective* reproductive ratio, *R_eff_*, is thus a time-dependent function for the average number of infected cells produced by an average infected cell at a given point, t, in time. *R_eff_* generalizes *R_0_*, the *basic* reproductive ratio, which evaluates the average infectivity only at the initial time point.

Solving the necessary and sufficient conditions for an R5-to-X4 switch, *d/dt*(V_4_(t*))>*d/dt*(V_5_(t*)) and V_4_(t*) = V_5_(t*), we see that a switch occurs in Model 3 if and only if:
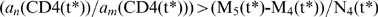
(4)But a_m_>a_n_ for all time, so, in particular, at the switch time t* we have a_n_(CD4(t*))/a_m_(CD4(t*))<1. The right-hand side of Equation (4) must therefore be less than 1, meaning that at the switch point N_4_(t*)+M_4_(t*)>M_5_(t*). Thus, at the switch point t* there are more X4-infected CD4^+^ T cells than R5-infected CD4^+^ T cells. This implies that X4 had a higher effective reproductive ratio at some earlier point, t**. However, *R_eff4_* (t**)>*R_eff5_* (t**) does not imply that *R_eff4_* (t)>*R_eff5_* (t) for all t>t**: Equation (3) implies that when *N* decreases faster than *M and* when the resulting decrease to *N/M* is less than the increase to *a_n_/a_m_, R_eff5_* increases relative to *R_eff4_* (i.e., *R_eff4_/R_eff5_* decreases). But the condition for steady-state coexistence of X4 and R5 is *R_eff4_ = R_eff5_*, so by enabling *R_eff5_* to rebound relative to *R_eff4_* post-switch, the dual-compartment nature of Model 3 makes coexistence possible.

We can grasp the switch threshold in (4) more easily by substituting in the particular equations of (1), yielding the following switch condition (where the right-hand side is positive):
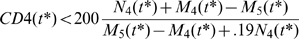
(5)Importantly, Equations (3) and (5) imply that, with the exception of changes to k_M5_, modulating parameters to accelerate CD4^+^ T cell decline hastens an R5 to X4 switch while changing parameters to mitigate CD4^+^ T cell decline hinders a phenotypic switch. Thus, successful antiretroviral therapy will generally inhibit X4's emergence. However, because R5 and X4 are now in competition, CCR5 inhibitors generate more complicated kinetics.

### CCR5 Inhibitors Can Accelerate X4 Emergence: The Need for CXCR4 Inhibitors or HAART

CCR5 inhibitors decrease *k_M5_*, causing R5's viral load to decline, and, as a result, memory CD4^+^ T cell counts to increase. The question we sought to answer is whether X4 infects sufficiently many of these R5-immune memory CD4^+^ T cells to counteract the increase in CD4^+^ T cells from CCR5 inhibition. We hypothesized that X4's ability to infect memory CD4 T cells would depend on *k_M4_*, and with a sufficiently large *k_M4_* (the “competitive regime”), X4 would infect a non-negligible fraction of newly R5-immune cells which and an increase in X4 would ensue. The temporal increase in X4 viral loads would thus cause greatly increased X4 infection of naïve CD4^+^ T cells, which yields accelerated naïve CD4^+^ T cell depletion. Indeed, numerical simulations (Text S1 contains more information on how these simulations were done) show that successful CCR5 blockage results in *accelerated* AIDS onset across much of parameter space (Figure 2C). This result does not change when Model 3 is extended to include the loss of virions due to the infection of new target cells (Figure S2). Importantly, the early immunodeficiency after effective CCR5 blockage is due to accelerating X4 emergence and increasing X4 viral loads as the efficacy of CCR5 inhibition increases in the “competitive regime” (Figure 2D).

Conversely, if *k_M4_* is sufficiently small (the “non-competitive regime”), X4 does not infect a sufficient number of dually-positive memory CD4^+^ T cells upon CCR5 blockage. This causes the uninfected memory CD4^+^ T cell population to increase during anti-CCR5 therapy, yielding a drop in *a_n_*/*a_m_* and hindering a potential switch to X4 as well as immunodeficiency (Figure 2C, small *k_M4_* regime). The latter result is to be expected from Model 2, because a weak *k_M4_* can be approximated by a complete lack of competition. Thus, a single parameter, *k_M4_*, controls the efficacy of anti-CCR5 therapy in dually infected HIV patients, highlighting the need for circumspection in prescribing these treatments.

**Figure 3:**
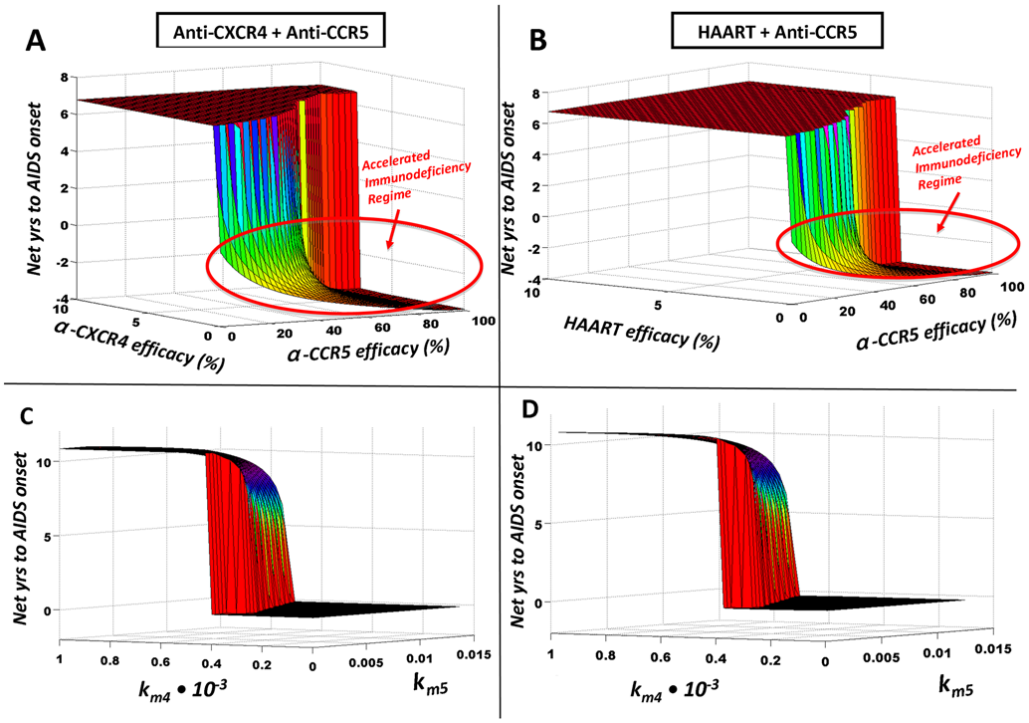
Combination treatment with HAART or CXCR4 inhibitors can prevent CCR5 inhibitors from accelerating AIDS-onset. (A) CXCR4 inhibitors with an efficacy of at least 5% prevent CCR5 inhibitors from accelerating time to AIDS in the “competitive regime.” Relative to no treatment, the net time to AIDS onset is positive (i.e. AIDS onset is never accelerated) for any CCR5 inhibitor accuracy if CXCR4 inhibitors have an accuracy of at least 10%. (B) Similarly, HAART with an efficacy of at least 7% prevents CCR5 inhibitors from accelerating time to AIDS (i.e., having a negative net time) in the competitive regime. (C) Net time to AIDS with dual-treatment using CCR5 and CXCR4 inhibitors, each with 80% efficacy, across different values of *k_M4_* and *k_M5_* relative to no treatment. The net time to AIDS onset is never negative for dual treatment with CCR5 and CXCR4 inhibitors (i.e. AIDS onset never accelerated) when the inhibitors have strong efficacies. (D) The net effect of dual-treatment with CCR5 inhibitors and HAART, each at 80% efficacy, on the time to AIDS at different values of *k_M4_* and *k_M5_*. The net time to AIDS onset is never negative for dual treatment with CCR5 inhibitors plus HAART (i.e. AIDS onset never accelerated) when the inhibitors and HAART have strong efficacies.

Given that CCR5 inhibitors accelerate R5-to-X4 switching and immunodeficiency across the wide swath of parameter space in which *k_M4_* is relatively large, the question arises as to whether CCR5 inhibitors are similarly deleterious when used in conjunction with CXCR4 inhibitors, which reduce *k_M4_*. Simulations show that adding a CXCR4 inhibitor with an efficacy of at least 5% is sufficient to prevent accelerated AIDS onset in the “competitive regime” (Figure 3A). Because X4 emergence is due to an increase in the relative fraction *a_n_*/*a_m_* of activated naïve to memory CD4^+^ T cells, we also simulated whether a generic antiretroviral therapy such as HAART, which increases CD4^+^ T cell counts and reduces *a_n_*/*a_m_*, also prevents the accelerated X4 emergence that CCR5 inhibitors can engender. The results of dual-treatment with HAART and CCR5 inhibitors are analogous to those shown for dual-treatment with CXCR4 and CCR5 inhibitors, proving that a relatively modest additional HAART therapy (with an efficacy above 7%) obviates the risk of CCR5 inhibitors accelerating immunodeficiency in the “competitive regime” (Figure 3B). Finally, generalizing across *k_M4_* and *k_M5_* parameter space shows that when treatment efficacies are sufficiently strong (e.g. 80% efficacies) dual treatment with CXCR4 inhibitors does not accelerate immunodeficiency relative to untreated individuals (Figure 3C). Similarly, dual-treatment with CCR5 inhibitors and HAART does not accelerate immunodeficiency relative to untreated individuals (Figure 3D).

## Discussion

Here we present a mathematical model of dual-strain R5 and X4 HIV *in vivo* dynamics and show that CCR5 inhibitors can accelerate the emergence of X4 virus and immunodeficiency. Two equivalent R5-to-X4 switch conditions were found: either the ratio of the relative fractions of activated naïve and memory CD4^+^ T cells (a_n_/a_m_) must surpass a threshold (Eq. 4) or, equivalently, CD4^+^ T cell counts must drop below a critical value (Eq. 5). The resultant “phenotypic” switch yields a drastic loss in CD4^+^ T cell counts, due to X4's depletion of R5-immune naïve CD4^+^ T cells. Of significant clinical importance, our results show that, across much of parameter space, CCR5 inhibitors may force an *early* switch to X4 virus, greatly accelerating CD4^+^ T cell depletion and AIDS onset. However, CCR5 inhibitors do not appear to have the deleterious effect of accelerating X4 emergence and immunodeficiency when they are used in conjunction with CXCR4 inhibitors or HAART.

The result that CCR5 blockers alone may promote X4 emergence is supported by data from a study on dually-infected *rhesus macaques* injected intravenously with the CCR5 inhibitor CMPD 167 [19]. After beginning treatment, two out of three primates manifested a transient increase of several logs in X4 viral load, essentially canceling the decrease in R5 viral load. Moreover, a clerical error in a recent study on the effect of the CCR5 inhibitor *maraviroc* on R5-only patients resulted in a dually-infected patient mistakenly being included in the trial [33]. That patient saw no change in total viral load as the X4 viral load increased upon CCR5 inhibition [20].

While CCR5 inhibitors alone may accelerate X4 emergence and AIDS onset, there is still good reason to consider their utility as part of a multi-therapy cocktail. Recent clinical data from the MOTIVATE 1&2 trials show that CCR5 inhibitors together with optimized background therapy yield larger increases in CD4 counts and larger reductions in viral loads when compared with optimized background therapy alone [34]. Our model simulations strongly support this result, showing that across much of parameter space, employing CCR5 inhibitors together with HAART lengthens the time to AIDS when compared with the time to AIDS under HAART alone (Figure S3).

But even if CCR5 inhibitors are a helpful component in a diversified anti-HIV therapy, one has to wonder about the greater immunological cost associated with blocking this chemokine receptor. A recent meta-population analysis of West Nile Virus (WNV) prevalence in four US states found that CCR5Δ32 homozygotes are approximately four times more likely to develop symptomatic WNV as are those with the wild-type receptor [35]. Previous murine models have suggested a mechanism by which CCR5 confers protective advantage against symptomatic WNV: CCR5 may promote the transfer of leukocytes to a WNV-infected individual's brain, aiding in immune control of encephalitis [36]. CCR5's potential protective advantage against symptomatic WNV may also help explain why CCR5Δ32/Δ32 is relatively common (5–14%) among European Caucasian cohorts, but near absent in African populations [37], the latter being at a far greater risk of contracting WNV.

Additionally, it is important to consider the prospect that CCR5 inhibition may lead to HIV evolving to bind to an entirely new coreceptor during early infection. A precedent for the evolution of new lentiviral coreceptor tropisms exists: the SIV endemic to *red-capped mangabeys* (RCMs) can utilize CCR2b rather than CCR5 [38]. This is likely because a large percentage (estimated at over 80%) of RCMs are homozygous for a 24 base-pair deletion in the gene for CCR5, and CCR5Δ24/Δ24 cells cannot be transfected with R5 virus [38]. The ability of SIVrcm to use CCR2b occurs despite almost all other known SIVs utilizing CCR5 exclusively *in vivo* [5]. Δ24 appears to be an ancient deletion: it has been found in both *red-capped mangabeys* and *sooty mangabeys*, species which diverged more than 10,000 years ago [38]. It is therefore possible that in the long-run HIV may evolve entirely new coreceptor usages in response to coreceptor inhibition.

## Supporting Information

Figure S1 Model 2 Incorrectly Predicts Decreased X4 Levels After Anti-CCR5 Treatment
(3.00 MB TIF)

Figure S2 Including Virion Loss Due to the Infection of New Target Cells has No Effect on Accelerated Immunodeficiency (see Figure 2c)
(3.00 MB TIF)

Figure S3 Anti-CCR5 Treatment With HAART Works Better Than HAART Alone
(3.00 MB TIF)

Text S1 Supplementary Methods: How the Simulations were Done
(0.06 MB DOC)
