# Supplementary figures and images for: Accelerated Immunodeficiency by Anti-CCR5 Treatment in HIV Infection
Source: PLoS Comput Biol. 2009 Aug 14;5(8):e1000467. doi: 10.1371/journal.pcbi.1000467 (PMC2715863; doi:10.1371/journal.pcbi.1000467)

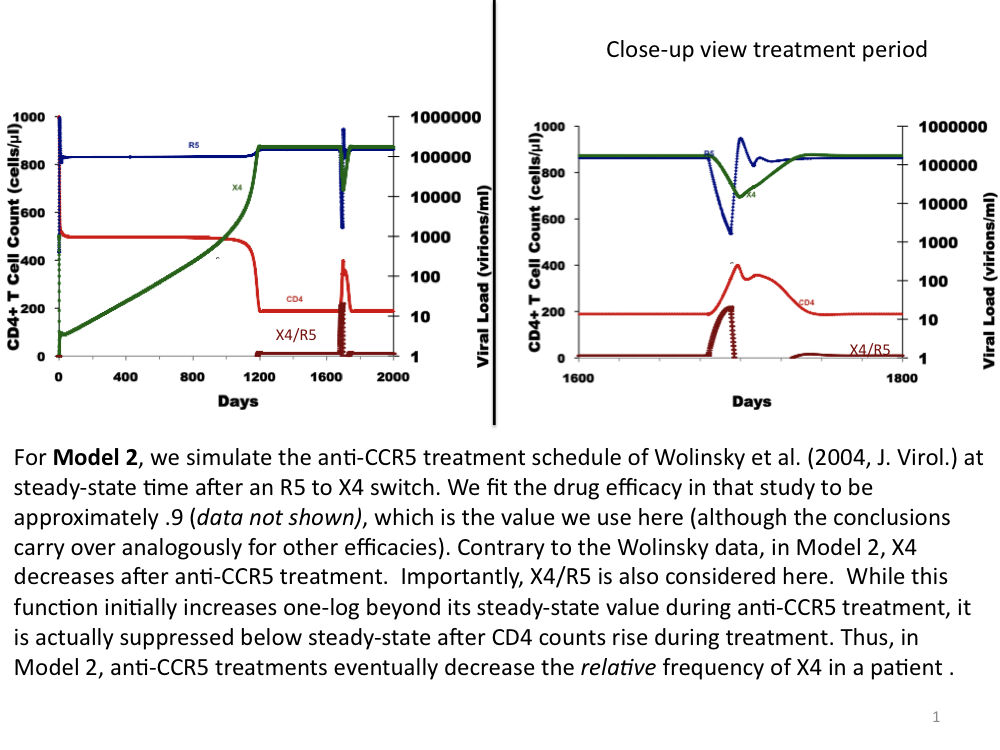

Supplement: Figure S1 — Model 2 Incorrectly Predicts Decreased X4 Levels After Anti-CCR5 Treatment (3.00 MB TIF) [file pcbi.1000467.s001.tif]

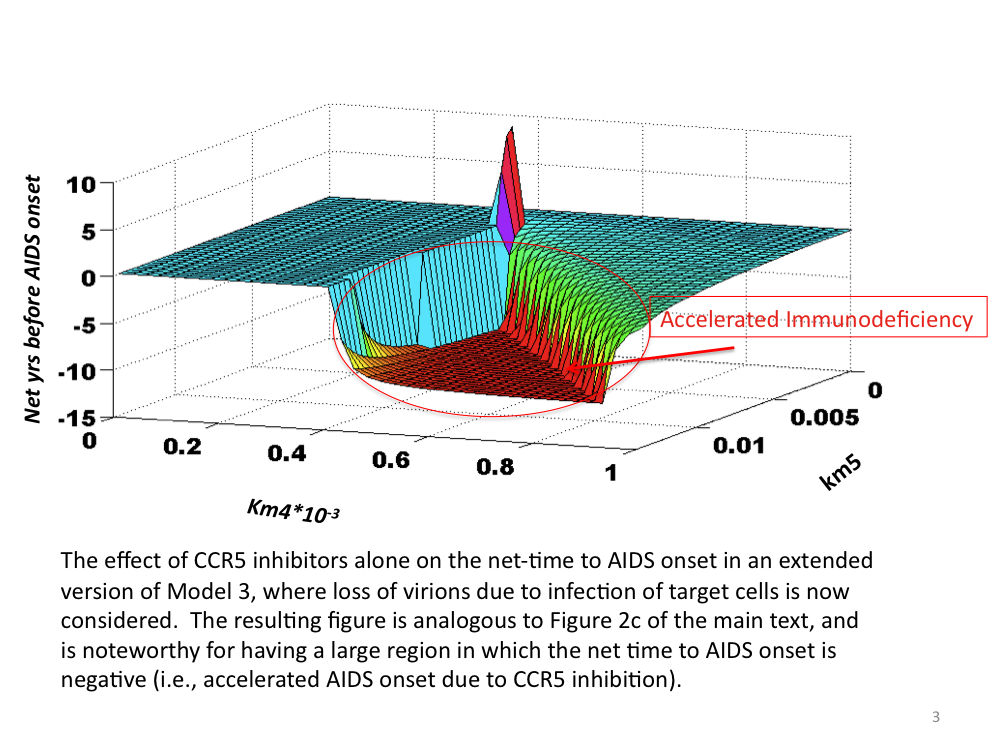

Supplement: Figure S2 — Including Virion Loss Due to the Infection of New Target Cells has No Effect on Accelerated Immunodeficiency (see Figure 2c) (3.00 MB TIF) [file pcbi.1000467.s002.tif]

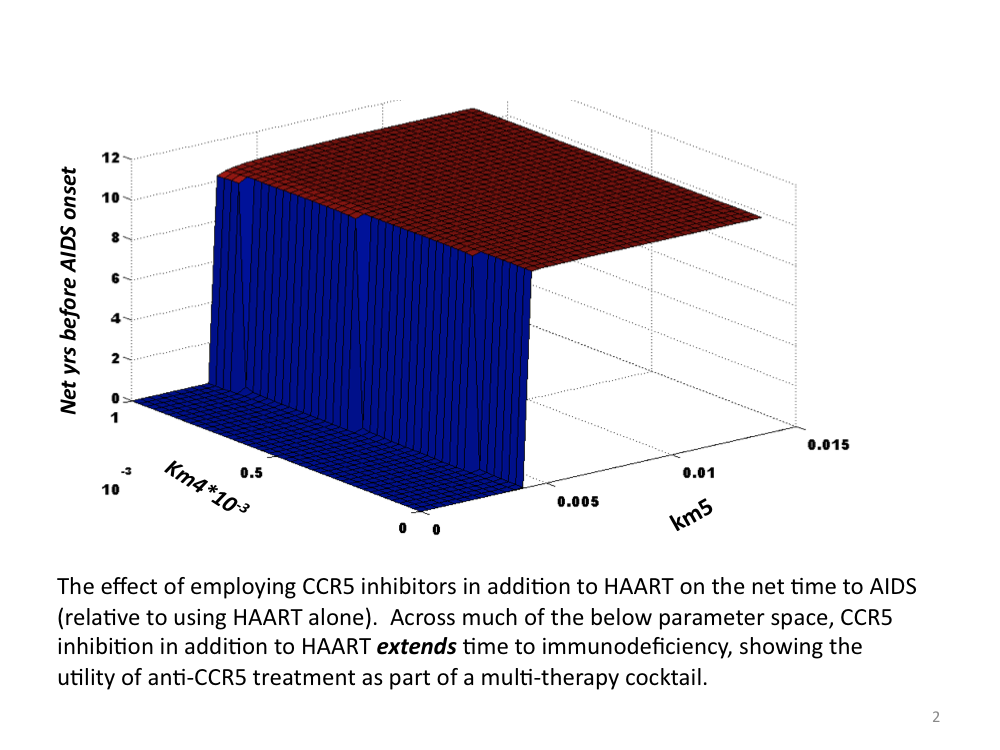

Supplement: Figure S3 — Anti-CCR5 Treatment With HAART Works Better Than HAART Alone (3.00 MB TIF) [file pcbi.1000467.s003.tif]
