# Supplementary material for: Accelerated Immunodeficiency by Anti-CCR5 Treatment in HIV Infection
Source: PLoS Comput Biol. 2009 Aug 14;5(8):e1000467. doi: 10.1371/journal.pcbi.1000467 (PMC2715863; doi:10.1371/journal.pcbi.1000467)
Supplement: Text S1 — Supplementary Methods: How the Simulations were Done (0.06 MB DOC) [file pcbi.1000467.s004.doc]

**Methods**

The Models 1-3 were first solved numerically using the program Berkeley Madonna. We applied the Rosenbruck, stiff algorithm with the parameters given in the Figures. Other than k_N4_, k_M4_, k_M5_ and f, these parameters have been estimated from *in vivo* measurements. Since the purpose of the first two models is to motivate the added complexity in Model 3 and since our main conclusions are taken from Model 3, we offer a justification of the parameter values for Model 3. In particular, *λ*, the rate at which naïve CD4^+^ T cells emigrate from the thymus, has been shown to remain relatively constant during HIV infection [1]. Following a recent theoretical analysis [2], we set *λ* to the constant value of 33 cells/(µl•day). The viral clearance rate, c, has been directly measured to have an average value of 23/day [3]. P, the rate of virion production by productively infected cells, is set to 2100/day, which is line with the in vivo measures in [4] and [5]. Finally, we set the infected cell death rate δ to 0.5/day, following the measurements in [6]. The final four parameters k_N4_, k_M4_, k_M5_ and f have unknown values, but can nonetheless be substantially restricted. F, the fraction of naïve CD4^+^ T cells that are activated by Ag-TCR interaction, is a probability and thus must be between 0 and 1. Furthermore, the FACS data of [7], leads us to restrict the infection rate coefficients as follows: k_N4_ >> k_M4_, k_M5_ >> k_M4_. We chose exact values for these 4 parameters, subject to the above constraints, by repeated simulations of our Models so as to produce the general dynamics of long-run HIV infection, including the common phenotypic switch.

We also note that because simulations require an exact form for a_n_ and a_m_, we used the particular form fit in (1). Of course, the analysis throughout this paper shows that we can apply any equations that satisfy (2), with obvious parameter adjustments.

Subsequent to these simulations, we reproduced our work in MATLAB (with the stiff ODE solvers, ode15s and ode23s) so that we could generate three-dimensional plots and show that the switch is *accelerated* when CCR5 is blocked in “competitive” regimes (i.e., those setups in which k_M4_ is relatively large). All code is available upon request.

**References**

1. Hazenberg MD, Otto SA, Cohen Stuart JW, Verschuren MC, Borleffs JC, et al. (2000) Increased cell division but not thymic dysfunction rapidly affects the T-cell receptor excision circle content of the naive T cell population in HIV-1 infection. Nat Med 6: 1036-1042.

2. Weinberger LS, Schaffer DV, Arkin AP (2003) Theoretical design of a gene therapy to prevent AIDS but not human immunodeficiency virus type 1 infection. J Virol 77: 10028-10036.

3. Ramratnam B, Bonhoeffer S, Binley J, Hurley A, Zhang L, et al. (1999) Rapid production and clearance of HIV-1 and hepatitis C virus assessed by large volume plasma apheresis. Lancet 354: 1782-1785.

4. Haase AT, Henry K, Zupancic M, Sedgewick G, Faust RA, et al. (1996) Quantitative image analysis of HIV-1 infection in lymphoid tissue. Science 274: 985-989.

5. Chen HY, Di Mascio M, Perelson AS, Ho DD, Zhang L (2007) Determination of virus burst size in vivo using a single-cycle SIV in rhesus macaques. Proc Natl Acad Sci U S A 104: 19079-19084.

6. Perelson AS, Neumann AU, Markowitz M, Leonard JM, Ho DD (1996) HIV-1 dynamics in vivo: virion clearance rate, infected cell life-span, and viral generation time. Science 271: 1582-1586.

7. Lee B, Sharron M, Montaner LJ, Weissman D, Doms RW (1999) Quantification of CD4, CCR5, and CXCR4 levels on lymphocyte subsets, dendritic cells, and differentially conditioned monocyte-derived macrophages. Proc Natl Acad Sci U S A 96: 5215-5220.
